# Supplementary material for: MetaRibo-Seq measures translation in microbiomes
Source: Nat Commun. 2020 Jun 29;11:3268. doi: 10.1038/s41467-020-17081-z (PMC7324362; doi:10.1038/s41467-020-17081-z)
Supplement: Supplementary file 10 — Supplementary Data 7 [file 41467_2020_17081_MOESM10_ESM.zip › File2/Confidence_VeryHigh_Taxonomy/136129_out.krona.html]

Javascript must be enabled to view this page.

members
magnitude
magnitudeUnassigned
count
unassigned
taxon
rank

136129\_out

15

15
superkingdom
2

15
1239
phylum

class
186801
12

12
order
186802

7
186803
family

SRS075773\_contig\_number\_contig-100\_3661.219377
1

28050
genus
6

6

SRS015431\_contig\_number\_81878SRS048164\_contig\_number\_6473SRS051882\_contig\_number\_13422SRS148721\_contig\_number\_52883SRS149879\_contig\_number\_6355SRS893378\_contig\_number\_15994
446043
species

family
541000
1

1

SRS015486\_contig\_number\_1685
genus
1263

1715004
species

SRS050422\_contig\_number\_contig-100\_12638.12638SRS098644\_contig\_number\_21517SRS147346\_contig\_number\_contig-100\_1703.332773
3

1897044
species
1

SRS051031\_contig\_number\_contig-100\_14324.14324

species
1897035

SRS013965\_contig\_number\_contig-100\_4946.227863SRS024388\_contig\_number\_contig-100\_13526.74241SRS049896\_contig\_number\_contig-100\_667.169449
3
